# Formation of Alginate/Chitosan Interpenetrated Networks Revealed by EPR Spectroscopy

**DOI:** 10.3390/gels9030231

**Published:** 2023-03-16

**Authors:** Jean-Patrick Joly, Ludmila Aricov, George-Alin Balan, Elena Irina Popescu, Sorin Mocanu, Anca Ruxandra Leonties, Iulia Matei, Sylvain R. A. Marque, Gabriela Ionita

**Affiliations:** 1 Aix Marseille University, CNRS, Institut de Chimie Radicalaire, UMR 7273, Case 551, Avenue Escadrille Normandie-Niemen, CEDEX 20, 13397 Marseille, France; 2 “Ilie Murgulescu” Institute of Physical Chemistry of the Romanian Academy, 202 Splaiul Independentei, 060021 Bucharest, Romania

**Keywords:** IPN, spin-labeled alginate, spin-labeled chitosan, EPR spectroscopy, rheology, IR spectroscopy

## Abstract

This study analyzes the physico-chemical properties of interpenetrated polymer networks (IPNs) and semi-IPN resulting from cross-linking chitosan with glutaraldehyde and alginate with Ca^2+^ cations, as a function of the order in which the cross-linking agents are added to the polymer mixture. Three physico-chemical methods were used to assess the differences between systems: rheology, IR spectroscopy, and electron paramagnetic resonance (EPR) spectroscopy. While rheology and IR spectroscopy are commonly used to characterize gel materials, EPR spectroscopy is rarely used, but has the advantage of providing local information about the dynamics of a system. The rheological parameters, which describe the global behavior of the samples, show that semi-IPN systems have a weaker gel behavior and the order of introducing the cross-linker in the polymer systems plays a role. The IR spectra of samples resulting by adding only Ca^2+^ or Ca^2+^ as the first cross-linker are similar to that of the alginate gel, while the spectra of samples in which glutaraldehyde is firstly added resemble the chitosan gel spectrum. Using spin-labeled alginate and spin-labeled chitosan, we monitored the changes occurring in the dynamic of the spin labels due to the formation of IPN and semi-IPN. The results show that the order of adding the cross-linking agents influences the dynamic of the IPN network, and that the formation of the alginate network determines the characteristics of the entire IPN system. The EPR data were correlated with the rheological parameters and IR spectra of the analyzed samples.

## 1. Introduction

The term interpenetrated polymer network (IPN) refers to polymeric materials that result from the interpenetration of two or more polymer networks that are not covalently connected to each other, but can only be separated by the breaking of chemical bonds [1,2,3,4]. Thus, a mixture of two polymers does not represent an interpenetrated network. The association of the two polymers arises from a combination of electrostatic forces, hydrogen bonding, hydrophobic interactions, and/or van der Waals forces, and, as such, the formation of the IPN network can strongly alter the physico-chemical properties of the solution. These include the solubility, mechanical properties, the permeability, and the electric conductivity. The properties of an IPN network are not a simple average of the properties of the constituting polymers, but depend on the polymers type and on the cross-linking method applied [5]. By varying these, new systems with improved properties, often substantially different from those of the constituting polymers, can be obtained. Therefore, a deeper understanding of the molecular interactions that occur at the interfaces in IPN polymer networks would allow tailoring of their properties to create more adaptable soft materials.

Polysaccharides are a class of compounds that can be part of IPN networks to form new systems whose properties can be controlled by changing the concentration of polysaccharide or cross-linking agent. The IPN networks resulting from the combination of natural or biocompatible polymers are, in many cases, sensitive to environmental changes and can ensure the optimization of the release control of some drugs, as well as proteins [5,6] and even cells [7]. When only the network of one of the polymers is cross-linked, a semi-IPN is formed. Obtaining IPN networks can be achieved by two methods: sequential (to the solution of a polymer, the cross-linking agent is added, after which the second polymer solution is added, followed by the cross-linking agent for the second); and simultaneous (to the solution of the two polymers, the two cross-linking agents are added together) [1].

**Figure 1:**
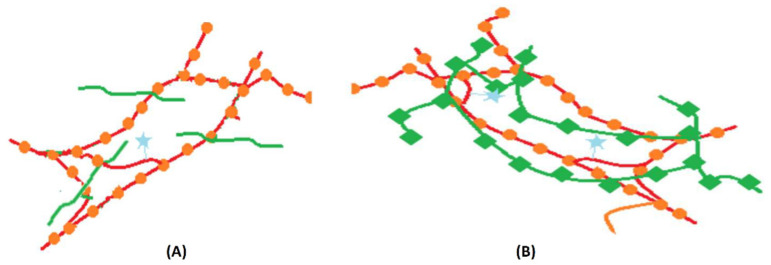
Representations of a semi-IPN network (**A**) and an IPN network (**B**). Circles and squares represent the cross-linking points of each polymer. The spin label is marked by a star.

In Figure 1, IPN and semi-IPN networks are schematically represented. As observed, there are connections of a covalent nature only between polymer chains of the same type. In this study, we used polysaccharides alginate and chitosan, which can lead to new biocompatible systems. The literature presents a significant number of examples for IPN and semi-IPN networks that include either alginate or chitosan [8]. In the case of alginate, the cross-links are determined by the complexation of the carboxyl groups of the guluronic blocks with Ca^2+^ cations [9]. Chitosan has amino groups in its structure that can react with glutaraldehyde, used as cross-linking agent, which leads to gel formation [10].

Recent years have seen an increased interest in alginate/chitosan systems due to their emerging applications in various areas, including the pharmaceutical and food industries for the encapsulation and controlled release of biologically active substances [11], in medicine for tissue engineering [12,13,14,15], and in the environmental sciences for waste-water remediation [16,17].

The conventional methods used to characterize IPN networks include techniques such as: IR spectroscopy, rheology, electron microscopy techniques, and calorimetry [3]. We reason that EPR spectroscopy can be a useful method to prove that different polysaccharide chains are not cross-linked. For this, we used spin-labeled alginate and spin-labeled chitosan. The present study is underpinned by previous EPR studies on different hydrogels containing polysaccharides or oligosaccharides. For instance, by using spin-labeled cyclodextrins, it was possible to monitor the formation of a polymeric gel resulting from the reaction of isocyanato-end-capped polyethylene glycol or polypropylene glycol with β-cyclodextrin, and to obtain information on the reactants [18]. We also studied the accessibility of gels of a series of spin probes with molecular weight covering the scale of 200–60 kDa, and the spin probe encapsulation properties of various gels [19]. By using spin-labeled alginate, it was possible to evidence the formation of alginate gels in the presence of various divalent cations and the diffusion of both polymer and cations through the bulk of the gel [20]. Using the same method, we demonstrated that, by appending host and guest units to alginate gels, the gel properties can be modified, as the rheological and EPR data have shown [21]. Similarly, we used EPR spectroscopy to evidence the host–guest interactions in solutions of polyacrylic acid functionalized with alkyl chains or adamantyl (guest) moieties and alginate functionalized with cyclodextrin (host) units. We found that the host–guest interactions between the appended units diminish the repulsive forces between the anionic polymers and influence the formation of inter-coils of polyacrylic acid [22]. By using the same approach, in this study, we aim to link the local information provided by EPR measurements with the overall properties of the alginate/chitosan systems that can be obtained by rheological measurements and IR spectroscopy.

## 2. Results and Discussion

The systems analyzed in this study by rheology, IR spectroscopy, and EPR spectroscopy are described in Table 1. Stock solutions of 1% chitosan and 1% alginate were prepared and then mixed in equal volumes. Spin-labeled alginate or spin-labeled chitosan to ensure an 0.1% concentration were added to the systems described in Table 1 in order to evidence the changes in spin label (TEMPO moiety, 2,2,6,6-tetramethyl-1-piperidinyloxy) dynamic after the addition of cross-linking agents.

For the alginate/chitosan mixture, three experiments were carried out: one in which the calcium salt was added first, followed by glutaraldehyde (sample 4); the second in which glutaraldehyde was added first, then the calcium salt (sample 5); and the third in which both cross-linkers were added simultaneously (sample 6). Figure S1 shows images of the analyzed systems. For samples 3 and 7 that have glutaraldehyde as cross-linker only, it can be observed that gelation occurs in the entire mass of the systems. For samples that result by adding Ca^2+^ cations as cross-linker (independently of the presence of glutaraldehyde), the resulting gels are separated from solution.

### 2.1. Rheological Properties of Alginate/Chitosan Systems

**Figure 2:**
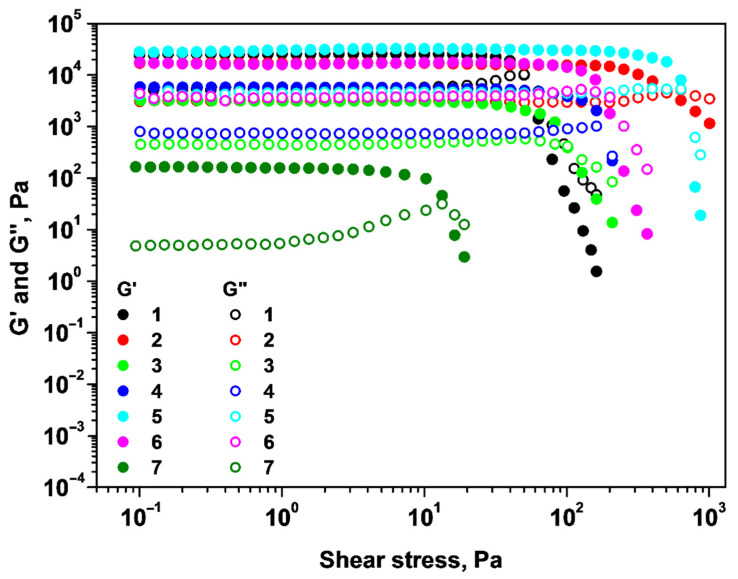
Dependence of storage and loss moduli on shear stress for samples 1–7.

The linear viscoelastic region of these materials was investigated and the results are presented in Figure 2. The storage modulus (at a shear stress of 0.1 Pa) and yield stress parameter (determined by tangent analysis) of the samples 1–7 are illustrated in Table 2.

As can be observed from Figure 2, the storage modulus (G′) is greater than the loss modulus (G″) in the linear viscoelastic region (LVER) for the studied systems. After the linear region, G′ begins to decrease, indicating the elastic limit (yield stress) and the beginning of plastic deformation of the systems. High yield stress and the high G′ values, as well as large LVER, indicate stiffer materials that are more resistant to deformation [23]. The rheological response of samples 1–7 depends on the type of cross-linkers introduced in the systems and also on the order in which these are added. Consequently, system 5 presents the best rheological performance toward deformation, unlike system 7, which has the weakest performance. It can also be noted that the presence of alginate in sample 3 increases the G′ with an order of magnitude compared with the chitosan gel (sample 7). Rheological measurements indicate that the semi-IPN alginate/chitosan systems (samples 2 and 3) have weaker gel properties compared with the alginate gel.

**Figure 3:**
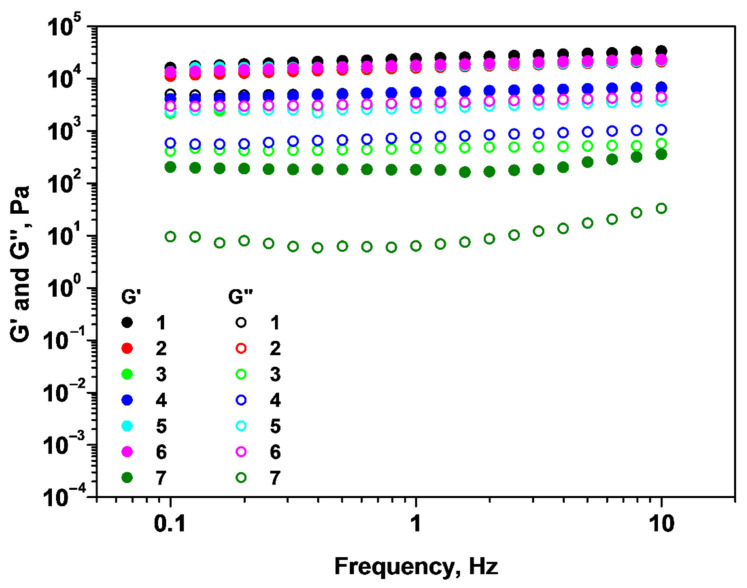
Dependence of storage and loss moduli on oscillation frequency for samples 1–7.

The viscoelastic fingerprint of these materials was studied and the results are summarized in Figure 3.

The systems 1–7 have a gel-like behavior, as is revealed by the G′ and G″ dependence on the applied frequency range. For instance, on this frequency interval, G′ is greater than G″, proving that the viscoelastic materials have good tolerance to external forces. Indicators of the strength of a gel are high values of G′ and G″, and also frequency independence of G′ [24,25]. As a result, we can say that systems 1, 2, 5, and 6 are the most resistant gels, as indicated by higher rheological moduli values and independence of the storage modulus (G′) on the applied frequency. In contrast to system 7, which is the weakest gel (lowest G′ and G″), systems 3 and 4 are gels with reasonable strength and comparable viscoelastic moduli.

**Figure 4:**
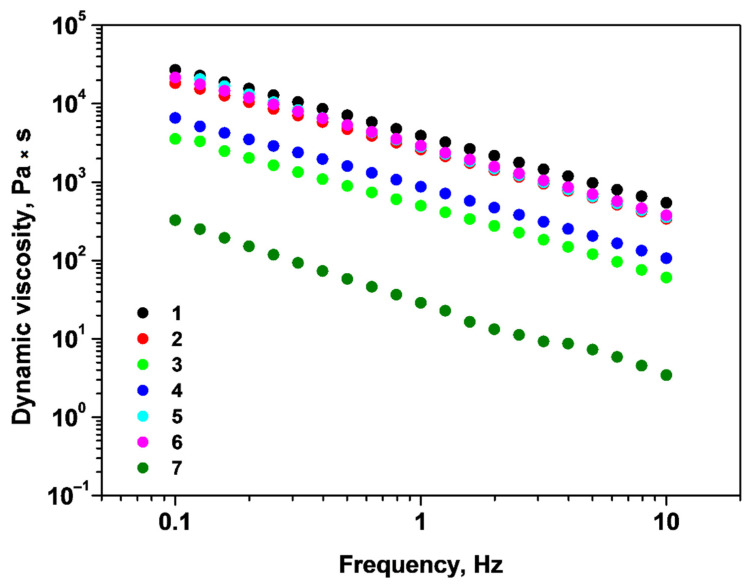
Dynamic viscosity as a function of oscillation frequency for samples 1–7.

The dependence of dynamic viscosity on the applied frequency was also investigated, and the obtained curves are presented in Figure 4. For all systems, the dynamic viscosity decreases with the oscillation frequency, pointing to a non-Newtonian pseudo-plastic nature. The highest viscosity and comparable flow curves are found for samples 1, 2, 5, and 6, indicating that the addition of Ca^2+^ cations enhances the viscosity parameter. Systems 3 and 4 have intermediate viscosity values with low difference between them, therefore, the addition of Ca^2+^ prior to glutaraldehyde has no appreciable effect on the flow parameter. The lowest viscosity is noticed for sample 7. These findings are consistent with the amplitude and frequency sweep stress results.

### 2.2. Characterization by IR Spectroscopy

**Figure 5:**
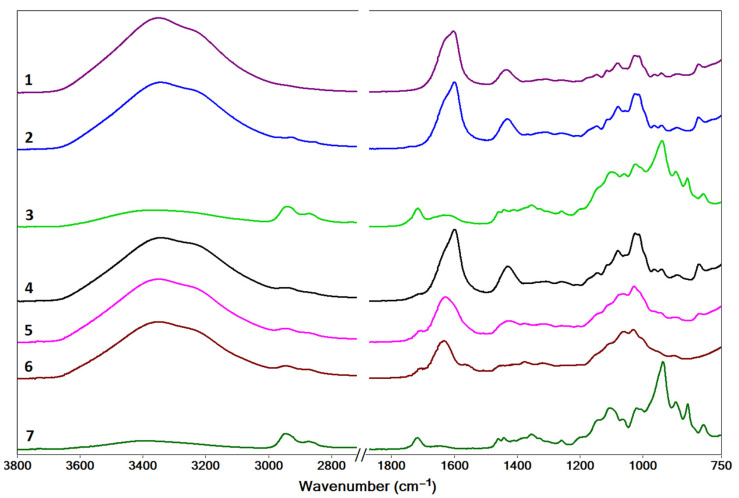
The IR spectra of isolated and dried gel networks for samples 1–7.

The IR spectra of dried samples of systems 1–7 are shown in Figure 5. They display vibrational modes characteristic to the structural groups in the polysaccharide units that have been assigned according to data from the literature for alginate [26,27,28] and chitosan [29,30,31], and are given in Table S1.

For the alginate gel prepared in the presence of Ca^2+^ ions (sample 1), three spectral regions of interest are evidenced: (i) 3600–3000 cm^−1^, comprising stretching vibrations of hydroxyl groups, ν(O-H); (ii) 1700–1400 cm^−1^, revealing two sharp bands at 1603 cm^−1^ and 1425 cm^−1^ corresponding to the asymmetric, ν_a_(COO^−^), and symmetric, ν_s_(COO^−^), stretching of carboxylate groups, respectively; (iii) 1200–800 cm^−1^ (includes the fingerprint region 950–750 cm^−1^ [26]), corresponding to C-O, C-C, and C-O-C stretching and deformation vibrations in the mannuronic and guluronic rings.

For the chitosan gel formed by cross-linking with glutaraldehyde (sample 7), the main spectral regions are: (i) 3650–3100 cm^−1^, comprising overlapping stretching vibrations of the hydroxyl, ν(O-H) and amino, ν(N-H), groups; (ii) 2950–2700 cm^−1^, corresponding to stretching vibrations of the aliphatic C-H groups, ν(C-H); (iii) a broad band centered at 1646 cm^−1^ arising from the superposition of the ν(C=N) stretching of the imine bond formed by cross-linking with glutaraldehyde [10,31] and the ν(C=O) stretching of residual N-acetyl groups in chitosan (amide I band) [31]; (iv) 1500–1200 cm^−1^, bending vibrations of the -CH_3_ and -CH_2_- groups, δ(C-H); and (v) 1150–800 cm^−1^, stretching and deformation vibrations associated with the C-O, C-O-C, C-C, and C-H skeletal vibrations of the saccharide structure. The absence of characteristic primary amine bending vibrational modes in the region 1590–1550 cm^−1^ indicates efficient cross-linking of chitosan by glutaraldehyde [32].

The IR spectra of samples 2 and 4 are similar to that of sample 1, which indicates that the initial addition of Ca^2+^ ions determines the gelation of alginate irrespective of the presence of chitosan in the system. The spectra of samples 7 and 3 also resemble each other, with the exception of a broadening and shift to lower wavenumbers of the band at 1646 cm^−1^, which is observed at 1627 cm^−1^ in sample 3. This shift may indicate some degree of interaction between alginate and chitosan [33], as well as a change in the number/ratio of C=N and C=O bonds, indicative of the formation of fewer imine cross-links in the presence of alginate.

In samples 5 and 6, their spectra are very similar. While they are also quite similar to the spectrum of sample 4, some small differences can be noted. The asymmetric ν_a_(COO^−^) stretching of alginate (observed at 1603 cm^−1^ in sample 1 and 1599 cm^−1^ in sample 4) shifts to higher wavenumbers in samples 5 (1627 cm^−1^) and 6 (1633 cm^−1^), which may indicate a strengthening of these bonds. The band corresponding to symmetric ν_s_(COO^−^) stretching is less prominent as it is overlapped with the ν(C=N) band corresponding to the imine groups in samples 5 and 6. The spectral profile in the range 1100–1000 cm^−1^ is similar for the samples containing Ca^2+^/alginate gel. For all systems, the broad band at 3600–3000 cm^−1^, attributed to ν(O-H) and ν(N-H) vibrations from alginate and chitosan, is noticeable. The bands in the region 2950–2700 cm^−1^, attributed to ν(C-H) stretching vibrations, are present in all samples containing chitosan.

### 2.3. Nanoscale Features Revealed by EPR Spectroscopy

The EPR spectroscopy is a suitable method for the investigation of various soft materials systems, including gels, by analyses of the dynamics and distribution of paramagnetic species in microenvironments with different properties [34]. The information obtained by the EPR method can be of use for analyses of the transport properties of gel materials, of the interactions between the encapsulated compounds and the gel network, and can be a useful tool in designing and tuning the properties of such materials.

In order to monitor the formation of polysaccharide gel networks by EPR spectroscopy, we introduced either spin-labeled alginate or spin-labeled chitosan in samples 1–7. As shown previously, the sol–gel phase transition of spin-labeled alginate is accompanied by a change from a relatively fast motion in solution to a restricted motion in the gel phase [20].

**Figure 6:**
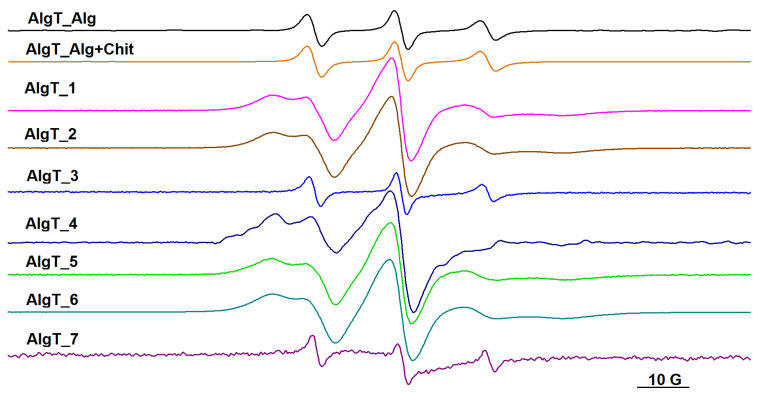
The EPR spectra of spin-labeled alginate (AlgT) in alginate solution, in a solution of alginate and chitosan, and in gel samples 1–7.

In alginate solution and in the solution containing the alginate/chitosan mixture, the EPR spectra of spin-labeled alginate (AlgT) are similar (see Figure 6, the EPR spectra AlgT_Alg and AlgT_Alg+Chit), revealing a dynamic in the quasi-isotropic regime. Based on this observation, we can conclude that in solution, these two polysaccharides do not interact, although they have in their structures functional groups that can interact with each other (alginate has carboxyl groups, while chitosan has amino groups). It can be noted that the value of the hyperfine coupling constant, a_N_, is not sensitive to the presence of chitosan in solution, which also sustains the idea that electrostatic interactions between the polysaccharide chains do not influence the polarity of the close environment of the spin label attached to the alginate chain.

**Figure 7:**
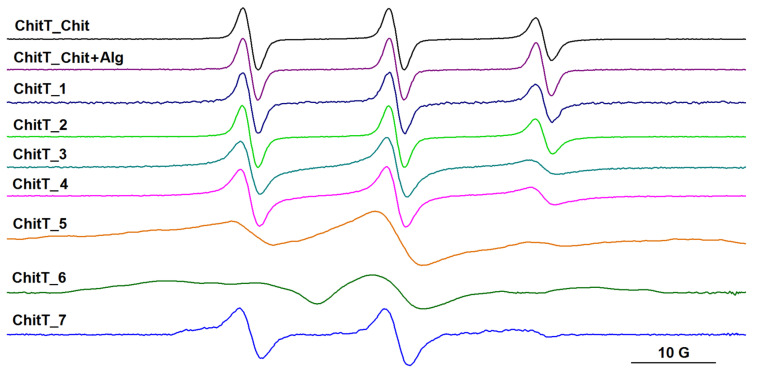
The EPR spectra of spin-labeled chitosan (ChitT) in chitosan solution, in a solution of chitosan and alginate, and in gel samples 1–7.

Depending on the type of cross-linkers used to generate the polysaccharide networks, we noticed the evolution of the spin-label dynamics by following the changes in the EPR spectra of either spin-labeled alginate (see Figure 6) or spin-labeled chitosan (see Figure 7).

In the absence of Ca^2+^ as cross-linker, the motion of spin-labeled alginate remains in a fast-motion regime, as can be noted in Figure 6, in the AlgT_3 spectrum. In the case of samples 3 and 7, which have only glutaraldehyde as cross-linker, a decrease in a_N_ is noticed, which indicates that the spin label attached to the alginate chain senses a less polar environment after the formation of the chitosan network. However, the dynamic of spin-labeled alginate remains in the quasi-isotropic regime, similar to the solutions of polysaccharides.

For samples that contain alginate and Ca^2+^ as cross-linker, the EPR spectra indicate a restricted motion (samples 2, 4, 5, and 6). These spectra are similar and their EPR parameters (rotational correlation time, τ, hyperfine coupling constant a_N_, isotropic, and 2A_zz_, anisotropic, parallel component) are indicated in Table 3. The parameters of the EPR spectra of spin-labeled alginate corresponding to samples 1, 2, and 4–6 were obtained by simulation with the NLSL program, considering two spectral components in each case [35].

As discussed above, all systems obtained by initial complexation of alginate with Ca^2+^ ions (samples 2, 3, 4–6) present a similar spectral pattern in the IR region. This trend is also observed in the case of the EPR spectra, as semi-IPN system 2 and IPN systems 4–6 show the spectral pattern of the alginate gel (sample 1). The mobile component is attributed to the spin labels attached to the alginate chain that are not involved in the complexation process with Ca^2+^ ions.

Spin-labeled chitosan was used to evidence the formation of the chitosan gel network. As expected, the presence of both cross-linkers leads to a higher degree of immobilization of the spin label (see Figure 7). The spectrum of spin-labeled chitosan in alginate gel (sample 1) is similar to that in sample 2, which corresponds to a semi-IPN based on the alginate/Ca^2+^ network, and the spectrum of spin-labeled chitosan in sample 4 is similar to that in sample 5. It can be noticed that, in the presence of both alginate and Ca^2+^, the spectral lines of spin-labeled chitosan become broader, which may indicate that the IPN network and chitosan network coexist in samples 4–6. The parameters of the EPR spectra of spin-labeled chitosan showing a rapid motion are presented in Table S2.

Each spin-labeled polysaccharide is sensitive mainly to the presence of the cross-linker specific for the polysaccharide parent, as is demonstrated by the EPR spectra presented in Figure 6 and Figure 7.

By comparing the rheological data, which show that the presence of Ca^2+^ as a cross-linker leads to stronger gels, with the EPR results, which indicate that the presence of Ca^2+^ leads to a restricted motion of spin-labeled alginate, we can conclude that, for the IPN systems, the formation of the alginate network determines the characteristics of the gel network for the entire system.

The EPR measurements confirm that the presence of both cross-linkers determines a higher immobilization of the polymers, thus, indirectly demonstrating the interconnection of alginate and chitosan networks.

## 3. Conclusions

The main conclusion of this study is that EPR spectroscopy can add to the information regarding the characterization of semi-IPN or IPN systems that is provided by classical methods such as rheology and IR spectroscopy. By introducing spin-labeled polymers in the systems that generate semi-IPN or IPN networks, we can highlight the differences of dynamics of paramagnetic groups covalently attached to polysaccharide chains between IPN or semi-IPN networks compared to simple networks of pure polysaccharide gels. The dynamic of each spin-labeled polysaccharide is sensitive mainly to the cross-linking process involving the parent polysaccharide. All physico-chemical methods show that the samples have different parameters as a function of the cross-linkers present and of the order in which they are added. The study is to be completed by morphological investigations of the analyzed systems. Moreover, this study can be continued with an investigation of the influence of other parameters: the ratio between the two polysaccharides, the quantity of cross-linker for chitosan, and the type of divalent cation that can act as cross-linker for the alginate chains. By shedding light on the molecular interactions that occur at the alginate/chitosan interfaces, semi-IPN and IPN systems with properties tailored for specific applications can be designed.

## 4. Materials and Methods

Low molecular weight chitosan, glutaraldehyde (25 wt.% in water), calcium chloride, and 4-carboxy-TEMPO were purchased from Aldrich (St. Louis, MO, USA). Low viscosity alginic acid sodium salt was purchased from Alfa Aesar (Ward Hill, MA, USA).

The synthesis of spin-labeled alginate was described previously [20]. Spin-labeled chitosan was obtained similarly, by reaction between the amino groups of the chitosan chain and 4-carboxy-TEMPO, in the presence of N-hydroxysulfosuccinimide (NHSS) and 1-ethyl-3-(3-dimethylaminopropyl)-carbodiimide hydrochloride (EDC). Spin-labeled chitosan was purified by dialysis after precipitation and washing with acetone.

Stock solutions of 1% alginate and 1% chitosan (pH 5) were prepared and mixtures were obtained by adding 1 mL of each. A stock solution of CaCl_2_ 1 M was prepared. In order to cross-link the alginate, 1 mL of the CaCl_2_ solution was added, and 0.2 mL of glutaraldehyde 25% in order to cross-link chitosan. The concentration of spin-labeled polysaccharides in systems 1–7 was 0.1%.

A Kinexus Pro Rheometer (Malvern, UK) was used to measure the rheological properties of alginate/chitosan systems. The temperature was maintained at 25 °C using a Julabo CF41 cryo-compact circulator (Seelbach, Germany). The samples were placed between a cone and plate geometry (10 mm diameter and 1° cone angle). The linear viscoelastic region (LVER) was identified using amplitude sweep tests at 1 Hz and 0.1–1000 Pa. The viscoelastic character was evaluated by frequency sweep tests at a constant shear stress from LVER in the range of 0.1–10 Hz. The dynamic viscosity was measured at frequency ranging from 0.1 to 10 Hz. The results were presented in logarithmic scale.

The FTIR spectra were collected on a Thermo Scientific Nicolet iS10 FT-IR spectrometer (Waltham, MA, USA). Wet samples of alginate/chitosan systems were freeze-dried on a glass surface in order to eliminate the high absorption by water that could mask the vibrational bands of the polysaccharide chains. A background spectrum was recorded and subtracted from the spectra of the samples.

The EPR spectra were recorded on an X-band JEOL FA100 spectrometer (Tokyo, Japan) at room temperature using the following settings: frequency modulation of 100 kHz, microwave power of 0.998 mW, sweep time of 240 s, modulation amplitude of 1 G, time constant of 0.1 s, and a magnetic field scan range of 100 G. The EPR spectra evidencing a restricted motion of paramagnetic centers were simulated using the NLSL program developed by Budil et al. [35]. For the EPR spectra evidencing a faster motion of the paramagnetic moiety, in the isotropic dynamic regime, the rotational correlation times, τ, were determined using the following equation:

(1) τ=6.51×10−10ΔH0h0h−112+h0h+11/2−2

where Δ*H*_0_ is the peak-to-peak width (in Gauss) of the central line, and *h*_−1_, *h*_0,_ and *h*_+1_ are the heights of the low, central, and the high field lines, respectively [36].

## Figures and Tables

**Table 1:** The composition of the samples studied and the corresponding crosslinker agents.

Sample	1	2	3	4	5	6	7
Composition	alginate	alginate chitosan	alginate chitosan	alginate chitosan	alginate chitosan	alginate chitosan	chitosan
Crosslinker	Ca^2+^	Ca^2+^	GA ^1^	(1) Ca^2+^ (2) GA	(1) GA (2) Ca^2+^	Ca^2+^ + GA	GA

^1^ GA—glutaraldehyde.

**Table 2:** Storage modulus (G′) and yield stress parameter of samples 1–7.

System	G′ (Pa)	Yield Stress (Pa)
1	24,470	51
2	16,890	493
3	3227	103
4	5826	166
5	28,230	633
6	17,530	158
7	164	13

**Table 3:** The EPR parameters of spin-labeled alginate (AlgT) in alginate solution, in a solution of alginate and chitosan, and in gel samples 1–7, and the percentage of the slow component for spectra showing a restricted motion.

Sample	τ_1_ × 10^10^ (s)	2a_N_ (G)	τ_2_ × 10^8^ (s)	2A_zz_ (G)	% of the Slow Component
AlgT_Alg	9.45	34.04	-	-	-
AlgT_Alg+Chit	9.97	34.04	-	-	-
AlgT_1	9.94	32.6	6.71	57.42	71.1
AlgT_2	9.94	32.6	6.71	57.42	72.1
AlgT_3	10.35	33.86	-	-	-
AlgT_4	10.3	32.7	6.28	56.36	90.0
AlgT_5	9.60	32.4	8.50	58.08	78.5
AlgT_6	9.60	32.4	7.20	57.18	75.7
AlgT_7	6.32	33.74	-	-	-

## Data Availability

Data are contained within the article and Supplementary Materials.

## Supplementary Materials

The following supporting information can be downloaded at: https://www.mdpi.com/article/10.3390/gels9030231/s1, Figure S1: Images of systems 1–7 investigated in this study; Table S1 [37]: Assignment of the main vibrational modes of alginate and chitosan gels; Table S2: The EPR parameters of ChitT in chitosan solution, in a solution of alginate and chitosan, and in gel samples 1–7.
