# Supplementary material for: Formation of Alginate/Chitosan Interpenetrated Networks Revealed by EPR Spectroscopy
Source: Gels. 2023 Mar 16;9(3):231. doi: 10.3390/gels9030231 (PMC10048464; doi:10.3390/gels9030231)
Supplement: Supplementary file 1 [file gels-09-00231-s001.zip › gels-2264983-supplementary.pdf]

gels

Supplementary Material

Formation of Alginate/Chitosan Interpenetrated Networks Revealed by EPR Spectroscopy

Jean-Patrick Joly [1], Ludmila Aricov [2,*], George-Alin Balan [2], Elena Irina Popescu [2], Sorin Mocanu [2], Anca Ruxandra Leonties [2], Iulia Matei [2], Sylvain R. A. Marque [1] and Gabriela Ionita [2,*]

[1] Aix Marseille University, CNRS, Institut de Chimie Radicalaire, UMR 7273, Case 551, Avenue Escadrille Normandie-Niemen, CEDEX 20, 13397 Marseille, France

[2] "Ilie Murgulescu" Institute of Physical Chemistry of the Romanian Academy, 202 Splaiul Independentei, 060021 Bucharest, Romania

* Correspondence: laricov@icf.ro (L.A.); gabi2ionita@yahoo.com or ige@icf.ro (G.I.)

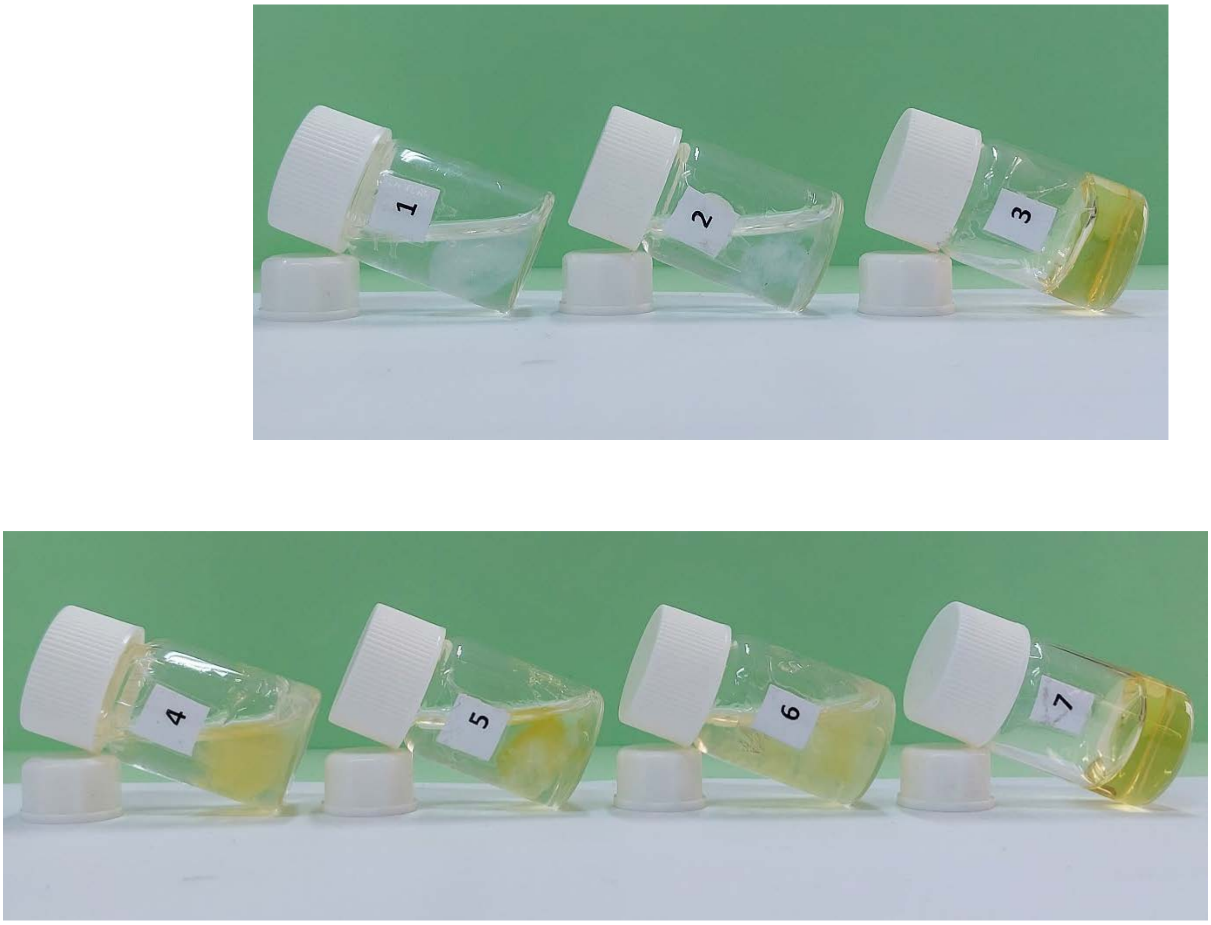

Figure S1. Images of systems 1-7 investigated in this study.

Table S1. Assignment of the main vibrational modes of alginate and chitosan gels.

Gel.	Wavenumber (cm^{-1}) [a]	Assignment [b]
alginate_Ca	3350 (s), 3240 (sh), broad	ν(O–H), hydrogen bonded
	1603 (s), 1625 (sh)	$\nu_a(COO^-)$
	1425	$\nu_s(COO^-)$
	1334	δ(C–C–H) + δ(O–C–H)
	1146; 1114 (w)	ν(C–O) + ν(C–C) + ν(C–OVC)
	1079; 1024, 1013	ν(C–O) + ν(C–C) + δ(C–C–C)
	963, 940 (w)	ν(C–O) + ν(C–C) + δ(C–C–O) (M+G)
	888 (w)	δ(C–H) (M+G)
	821	δ(C–C–O) + δ(C–C–H) (M)
	3390 (s), broad	ν(N-H), ν(O-H), hydrogen bonded
	2945, 2873	ν(C–H)
	1717	ν(C=O) unreacted GA [R1]
	1646, broad	ν(C=N) crosslink with GA +
		+ ν(C=O) residual acetyl (amide I)
chitosan_GA	1460, 1442	ν(C-N) + δ(C-H) in $-CH_3$
	1353	δ(C-H) in $-CH_2-$
	1258 (w)	ν(C-O-C)
	1198, 1187	δ(C-O-C)
	1144, 1104 (s), 1063, 1005, 1019, 936 (s),	ν(C-O) + ν(C-C) + ν(C-H) + ν($-CH_2$-OH)
	895, 857, 806	skeletal vibrations polysaccharide

Note: [a] s, strong; sh, shoulder; w, weak; [b] ν, stretching (a – asymmetric, s – symmetric); δ, deformation (bending); M – β-D-mannuronic acid; G – α-L-guluronic acid.

Table S2. The EPR parameters of ChitT in chitosan solution, in a solution of alginate and chitosan, and in gel samples 1–7.

Sample	T × 10^{10} (s)	$2a_N$ (G)
ChitT_Chit	2.28	34.17
ChitT_Alg+Chit	0.85	34.25
ChitT_1	3.30	34.30
ChitT_2	3.98	34.30
ChitT_3	17.8	34.25
ChitT_4	6.51	34.23
ChitT_7	32.75	33.92
